# Survey dataset for the perceived consciousness towards environmental sustainability by undergraduate students

**DOI:** 10.1016/j.dib.2022.107985

**Published:** 2022-02-22

**Authors:** Kevin Fuchs

**Affiliations:** Faculty of Hospitality and Tourism, Prince of Songkla University, 80 Moo 1 Vichitsongkram Road, Kathu District, Phuket 83120, Thailand

**Keywords:** Environmental sustainability, Intentions towards environmental sustainability, Tourism education

## Abstract

The scope of the dataset allows to examine undergraduate hospitality and tourism students’ intentions towards environmental sustainability. Moreover, it is possible to compare factors (knowledge, attitude, perceived behavioral control, and intention) towards environmental sustainability between hospitality and tourism students based on different socio-demographic characteristics. To fulfill these objectives, data was collected through a bilingual questionnaire containing 312 valid responses from undergraduate students studying towards a business degree. The questionnaire was administered at the Prince of Songkla University in Phuket, Thailand in the fourth quarter of 2021. Moreover, the data collection adhered to the NESH principles applicable for Social Science Research. The data collection instrument was validated for international consistency and reliability through a three-person panel of research experts and validated using the IOC method. Furthermore, the questionnaire was tested with a targeted sample consisting of ten students prior to its implementation. The dataset serves as an insightful reference for practitioners and policymakers in higher education to adjust their pedagogy, in addition to, as a secondary data source for educational researchers to examine undergraduate hospitality and tourism students’ intentions towards environmental sustainability.

## Specifications Table

**Table:** 

Subject	Social Sciences Social Science
Specific subject area	Examination of factors influencing intentions towards environmental sustainability of hospitality and tourism students in Thailand
Type of data	Table
How the data were acquired	Targeted online survey
Data format	Raw
Description of data collection	The data was collected through a bilingual questionnaire containing 312 valid responses from undergraduate students studying towards a business degree (majoring in hospitality and tourism). The questionnaire was administered at the Prince of Songkla University in Phuket, Thailand in the fourth quarter of 2021.
Data source location	• Prince of Songkla University • Phuket • Thailand
Data accessibility	The dataset has been deposited into an open repository and is available under the following permanent specifications: Repository name: Mendeley Data Data identification number: 10.17632/rgdj46688d.1 Direct URL to data: 10.17632/rgdj46688d.1

## Value of the Data

• The dataset allows examination of factors influencing intentions towards sustainability of hospitality and tourism students in Thailand.
• The dataset reveals correlations between factors (knowledge, attitudes, perceived behavioral control, and intentions) towards environmental sustainability between hospitality and tourism students.
• The dataset serves as an insightful reference for practitioners and policymakers in higher education to adjust their pedagogy.
• The dataset serves as secondary data source for educational researchers to examine undergraduate hospitality and tourism students’ intentions towards environmental sustainability.

## 1 Data Description

Environmental sustainability is a very articulated and complex concept that is widely discussed in many disciplines and many streams of the literature [1]. Increased awareness toward environmental sustainability manifests and leads to the adoption of the concepts to address environmental challenges, which played a key role in the birth of environmental movements [2]. Tourism is dependent upon the environment for much of its well-being [1,3]. Moreover, tourism education is at the forefront of impacting environmental sustainability in one way or another by educating tomorrow's tourism stakeholders [4]. The associated dataset can be interpreted for further analysis based on the tags and labels presented in Tables 1 and 2 underneath, as well as a copy of the survey can be found in the supplementary material.

**Table 1:** Description of the characteristics in the dataset.

Column	Data label	Explanation
Column *A*	Student Status	Degree student; Exchange student
Column *B*	Institution	Prince of Songkla University
Column *C*	Faculty	Faculty of Hospitality and Tourism; College of Computing; Faculty of International Studies
Column *D*	Gender	Male; Female; I do not wish to say; Other
Column *E*	Age Range	18–19 years old; 20–21 years old; 22–23 years old; 24 years or above
Column *F*	Year	Year 1; Year 2; Year 3; Year 4
Column *G*	Nationality	Thai; Foreign
Column *H*	Probe	Have you heard about environmental sustainability before? [Answer options: (Yes) and (No)].

**Table 2:** Questionnaire organized by their respective factor.

Column	Data label	Explanation
*Attitude*		

Column *I*	Question 1	In my opinion, it is important to protect the environment.
Column *J*	Question 2	I actively practice environmental sustainability at home (e.g., energy conservation, recycling).
Column *K*	Question 3	Everyone is responsible for caring for the environment
Column *L*	Question 4	I am concerned about the long-term future of the environment.
Column *M*	Question 5	In my opinion, it is important to conserve natural resources.
Column *N*	Question 6	I think that environmental sustainability is a waste of time and effort.
Column *O*	Question 7	I am a passionate advocate of environmental sustainability.

*Perceived behavioral control*		

Column *P*	Question 8	It is easy for me to perform environmentally sustainable activities (e.g., energy conservation, recycling).
Column *Q*	Question 9	I have control over my actions to support the environment.
Column *R*	Question 10	It is my decision whether or not to perform environmentally sustainable activities.
Column *S*	Question 11	I have the ability to carry out environmentally sustainable activities.
Column *T*	Question 12	I have control over performing environmentally sustainable activities.

*Intention*		

Column *U*	Question 13	I plan to increase environmentally sustainable activities (e.g., energy conservation, recycling) in the future.
Column *V*	Question 14	I intend to seek out more opportunities to be more environmentally active in the future.
Column *W*	Question 15	In the future, I plan to look into how I can play a greater role in protecting the environment.
Column *X*	Question 16	I do not expect to increase my level of support for the environment.

*Knowledge*		

Column *Y*	Question 17	I talk about the need to preserve the environment at home or with friends.
Column *Z*	Question 18	I have learned about sustainability in high school or university.
Column *AA*	Question 19	I am well informed about current issues that impact the environment.
Column *AB*	Question 20	I feel confident to talk about issues related to environmental sustainability.

## 2 Experimental Design, Materials and Methods

### 2.1 Sampling and collection

The data were collected from undergraduate students through simple random sampling, which is a probability sampling method that allows the sampling error to be calculated [5]. Etikan and Bala [6] noted that in social science research, it is advantageous to eliminate selection bias by the researcher or volunteer bias by the participant for representativeness of the results (p. 215). In the absence of an existing methodological frame, random sampling is deemed an appropriate method to apply for this study [5,6]. However, a disadvantage of random sampling is the lack of sufficient responses that fit the desired characteristic of interest. To manage such potential limitations, the questionnaire was administered in the final phase to specific socio-demographic clusters (i.e. gender or year of study) to increase the probability of reaching the desired population.

The questionnaire was administered with a bilingual option, i.e. Thai and English. Moreover, the questionnaire was administered at the Prince of Songkla University in Phuket, Thailand in the fourth quarter of 2021. After screening the collected data, 9 inconclusive/incomplete responses were discarded from inclusion. The sample size included was 312 to represent the population in data analysis. The confidence level of accurate sampling was estimated at 95% (*p* < 0.05), based on the total student enrolment and sample size that were included, and the margin of error was quantified as 5%. Based on eligible responses, the representative demographic profile in Table 3 summarizes the respondents’ gender, year of study, age range, nationality, and place of study (institution).

**Table 3:** Socio-demographic profile of the participants.

Characteristics	Frequency	Percentage (%)
*Gender*		
Female	231	74.1
Male	75	24.0
I do not wish to say	5	1.6
Other (not further specified)	1	0.3
*Year of Study*		
First Year	78	25.0
Second Year	89	28.5
Third Year	79	25.3
Fourth Year	66	21.2
*Age Range*		
18–19 years old	106	34.0
20–21 years old	169	54.2
22–23 years old	34	10.9
24 years or above	3	0.9
*Nationality*		
Thai	261	83.7
Foreign (not further specified)	51	16.3

### 2.2 Research instrument

The adaptation of seven (7) instead of five (5) points on the original Likert-type scale is described as a universal instrument to efficiently measure and evaluate the attitudes of respondents [7]. Fryer and Nakao [8] further added that gathering data to predict a population sample's sentiment on a particular issue is the primary advantage of this approach (p. 11). The default responses offer the participants seven answer options to express their satisfaction or dissatisfaction, with a neutral option at the midpoint. The questionnaire included a total of 28 attributes of which 8 were used to establish the socio-demographic profile and 20 to assess and evaluate the students’ attitudes. The default responses on the Likert-type scale ranged from 1 (lowest) to 7 (highest), i.e. Strongly Disagree (1), Disagree (2), Somewhat Disagree (3), Neither Agree or Disagree (4), Somewhat Agree (5), Agree (6), and Strongly Agree (7).

Moreover, the index of item-objective congruence (IOC) is a tool used to improve the validity and reliability of administered surveys and was used as the basis for screening the item quality. The survey was evaluated by three impartial research experts for content validity using an adapted version of the IOC index developed by Turner and Carlson [9]. The mean IOC score was 0.92, which is above the acceptable threshold of 0.80 [10]. Additionally, Cronbach's Alpha Coefficient (α) was calculated to “ensure consistency of test scores over different parts of the survey” [11]. The IOC (0.92) and previously reported Cronbach's Alpha (0.895) indicated high reliability for the research instrument that was used to collect the data. Lastly, the questionnaire was tested for comprehension with a limited sample (*n* = 10), although these responses were not included in the analysis.

## Ethics Statements

Institutional approval was obtained prior to collecting the data and before answering the survey, written informed consent was a precondition for participation. The participants were informed about the research and its purpose, that they had the right to withdraw at any stage, and that the data collected would be treated as confidential (i.e. anonymized in all reporting). For ethical considerations and to protect the identity of the participants, some specific information in the socio-demographic profile was generalized before disclosure in this paper, namely some specific minority nationalities were labeled as “foreign” instead of displaying the particular nationality as this could potentially allow exposing the identity of the participant. The foundation for ethical considerations is based on the principles formulated by the Norwegian National Research Ethics Committees [12].

## CRediT authorship contribution statement

**Kevin Fuchs:** Conceptualization, Methodology, Data curation, Writing – original draft.

## Declaration of Competing Interest

The authors declare that they have no known competing financial interests or personal relationships that could have appeared to influence the work reported in this paper.

## Appendix Supplementary materials

Image, application 1
