# Supplementary material for: Survey dataset for the perceived consciousness towards environmental sustainability by undergraduate students
Source: Data Brief. 2022 Feb 22;41:107985. doi: 10.1016/j.dib.2022.107985 (PMC8889337; doi:10.1016/j.dib.2022.107985)
Supplement: Supplementary file 1 [file mmc1.pdf]

สำรวจ / Survey

แบบสำรวจเกี่ยวกับความยั่งยืนด้านสิ่งแวดล้อม / Survey about Environmental Sustainability

In English below.

ขอขอบคุณนักศึกษาที่ยินยอมตอบแบบสำรวจเกี่ยวกับความคิดเห็นส่วนตัวของนักศึกษาที่มีต่อความยั่งยืนด้านสิ่งแวดล้อมนี้ด้วยความสมัครใจ แบบสำรวจชุดนี้มีอยู่ 2 ส่วน ส่วนแรกจะเกี่ยวกับข้อมูลทั่วไปของนักศึกษา และส่วนที่สองจะประกอบด้วยข้อความ 20 ข้อความที่มีคำตอบให้เลือกตอบเกี่ยวกับความคิดเห็นของนักศึกษาที่มีต่อความยั่งยืนด้านสิ่งแวดล้อม กรุณาตอบชุดคำถามต่อไปนี้ให้ตรงกับความคิดเห็นของนักศึกษามากที่สุด จะไม่มีการเปิดเผยคำตอบของนักศึกษาและข้อมูลจะถูกเก็บรวบรวมไว้สำหรับโครงการวิจัย หากนักศึกษามีข้อสงสัยเกี่ยวกับการสำรวจนี้ กรุณาติดต่ออาจารย์ Kevin [อีเมล: aj.kevin.fht@gmail.com] ขอขอบคุณอีกครั้ง! (ฉันอายุมากกว่า 18 ปีเพื่อดำเนินการต่อ)

Thank you for agreeing to voluntarily answer this survey about your personal view on environmental sustainability. There are two sections in this survey: The first section is about your profile and the second section includes 20 multiple-choice statements about your opinion on environmental sustainability. Please answer the following set of questions as honest as possible. Your response will be anonymous and the data will be collected for a research project. If you have any questions about the survey, please contact Aj. Kevin [e-mail: aj.kevin.fht@gmail.com]. Thank you again! (I am 18 years old older to proceed)

Next

Clear form

สำรวจ / Survey

(1) กรุณาให้ข้อมูลทั่วไปของนักศึกษา / Please provide a few general information about yourself

ก. ดิฉัน/ผมเป็นนักศึกษาระดับปริญญาตรีเต็มเวลาที่มหาวิทยาลัย Prince of Songkla University (a. I am a full-time undergraduate student at Prince of Songkla University) *

- ○ Yes
- ○ No

ข. ดิฉัน/ผมเป็นนักศึกษาของคณะต่อไปนี้ (b. I am a student of the following Faculty) *

Your answer

ค. เพศ (c. Gender) *

- ○ ชาย (Male)
- ○ หญิง (Female)
- ○ ขอไม่ตอบ (I do not wish to say)
- ○ Other:

ง. อายุ (d. Age) *

- ○ 18 – 19 ปี (18 - 19 years old)
- ○ 20 – 21 ปี (20 - 21 years old)
- ○ 22 – 23 ปี (22 - 23 years old)
- ○ 24 ปีขึ้นไป (24 years or above)

จ. ชั้นปี (e. Year of study) *

- ○ ปี 1 (Year 1)
- ○ ปี 2 (Year 2)
- ○ ปี 3 (Year 3)
- ○ ปี 4 (Year 4)
- ○ ปี 5 ขึ้นไป (Year 5 or above)

ฉ. ภาษาแม่หรือภาษาแรก (f. Native language) *

- ○ ภาษาไทย (Thai)
- ○ Other:

ช. เกรดเฉลี่ย (GPA) ปัจจุบันของนักศึกษา *** (g. What is your current GPA? ***) *

Your answer

***

- โปรดระบุเกรดเฉลี่ย (GPA) ปัจจุบันของนักศึกษา ให้ใส่ทศนิยม 2 ตำแหน่งหลังจุดทศนิยม ตัวอย่างเช่น 2.55 หรือ 3.63
- Please provide your current Grade Point Average (GPA) with two digits after the decimal point. For example, 2.55 or 3.63.

ซ. นักศึกษาเคยได้ยินเกี่ยวกับ "ความยั่งยืนด้านสิ่งแวดล้อม" มาก่อนหรือไม่? (h. Have you heard about "Environmental Sustainability" before?) *

- ○ ใช่ (Yes)
- ○ ไม่ (No)

กรุณาอ่านคำจำกัดความต่อไปนี้เกี่ยวกับ "ความยั่งยืนด้านสิ่งแวดล้อม" ก่อนที่นักศึกษาจะไปต่อในหน้าถัดไป (Please read the following definition about "Environmental Sustainability" before you move to the next page):

วิทยาศาสตร์สิ่งแวดล้อมเป็นเรื่องเกี่ยวกับการค้นหาวิธีการดำเนินชีวิตอย่างยั่งยืนมากขึ้น ซึ่งหมายถึงการใช้ทรัพยากรต่าง ๆ ในปัจจุบันในลักษณะที่รักษาทรัพยากรเหล่านั้นไว้เพื่ออนาคต ความยั่งยืนด้านสิ่งแวดล้อมไม่ได้หมายถึงการดำเนินชีวิตโดยปราศจากสิ่งฟุ่มเฟือย แต่เป็นการตระหนักถึงการใช้ทรัพยากรของคุณและการลดของเสียที่ไม่จำเป็น

Environmental science is all about finding ways to live more sustainably, which means using resources today in a way that maintains their supplies for the future. Environmental sustainability doesn't mean living without luxuries, but rather being aware of your resource consumption and reducing unnecessary waste.

Back | Next

Clear form

สำรวจ / Survey

aj.kevin.fht@gmail.com (not shared) Switch account

* Required

หลังจากให้ข้อมูลทั่วไปของนักศึกษาเสร็จเรียบร้อยแล้ว ตอนนี้ เรามาเริ่มต้นการสำรวจกันเลย! ในส่วนนี้ จะมีข้อความอยู่ทั้งหมด 20 ข้อความ และนักศึกษาจะมีตัวเลือกอยู่ 7 ตัวที่แตกต่างกันในการแสดงความคิดเห็นของนักศึกษาที่มีต่อแต่ละข้อความ ขอให้นักศึกษาตอบคำถามอย่างตรงไปตรงมา

The profiling section is now complete. Let's start with the survey! There will be a total of 20 statements and you have seven different options to express your opinion for each statement. Please answer them honestly.

1 = ไม่เห็นด้วยอย่างยิ่ง (Strongly Disagree)
2 = ไม่เห็นด้วย (Disagree)
3 = ค่อนข้างไม่เห็นด้วย (Somewhat Disagree)
4 = เป็นกลาง (Neither Agree or Disagree)
5 = ค่อนข้างเห็นด้วย (Somewhat Agree)
6 = เห็นด้วย (Agree)
7 = เห็นด้วยอย่างยิ่ง (Strongly Agree)

คำถาม 1-3 (Statement) 1-3

1.) ในความคิดของดิฉัน/ผม การปกป้องสิ่งแวดล้อมเป็นสิ่งสำคัญ (In my opinion, it is important to protect the environment) *

1 2 3 4 5 6 7

2.) ดิฉัน/ผมมีการปฏิบัติในเรื่องของความยั่งยืนด้านสิ่งแวดล้อมที่บ้านเป็นกิจวัตรอย่างแข็งขัน (เช่น การอนุรักษ์พลังงาน การรีไซเคิล) (I actively practice environmental sustainability at home [e.g., energy conservation, recycling]) *

1 2 3 4 5 6 7

3.) ทุกคนมีหน้าที่ในการดูแลสิ่งแวดล้อม (Everyone is responsible for caring for the environment) *

1 2 3 4 5 6 7

Back | Next

Clear form

สำรวจ / Survey

aj.kevin.fht@gmail.com (not shared) Switch account

* Required

1 = ไม่เห็นด้วยอย่างยิ่ง (Strongly Disagree)
2 = ไม่เห็นด้วย (Disagree)
3 = ค่อนข้างไม่เห็นด้วย (Somewhat Disagree)
4 = เป็นกลาง (Neither Agree or Disagree)
5 = ค่อนข้างเห็นด้วย (Somewhat Agree)
6 = เห็นด้วย (Agree)
7 = เห็นด้วยอย่างยิ่ง (Strongly Agree)

คำถาม 4-8 (Statement 4-8)

4.) ดิฉัน/ผมเป็นห่วงเกี่ยวกับอนาคตของสิ่งแวดล้อมในระยะยาว (I am concerned about the long-term future of the environment) *

1 2 3 4 5 6 7

5.) ในความคิดของดิฉัน/ผม การอนุรักษ์ทรัพยากรธรรมชาติเป็นสิ่งสำคัญ (In my opinion, it is important to conserve natural resources) *

1 2 3 4 5 6 7

6.) ดิฉัน/ผมคิดว่าความยั่งยืนด้านสิ่งแวดล้อมเป็นเรื่องที่เสียเวลาและเป็นความพยายามที่สูญเปล่า (I think that environmental sustainability is a waste of time and effort) *

1 2 3 4 5 6 7

7.) ดิฉัน/ผมหลงใหลในการให้การสนับสนุนความยั่งยืนด้านสิ่งแวดล้อม (I am a passionate advocate of environmental sustainability) *

1 2 3 4 5 6 7

8.) สำหรับดิฉัน/ผมแล้ว การปฏิบัติกิจกรรมต่าง ๆ แบบยั่งยืนด้านสิ่งแวดล้อม (เช่น การอนุรักษ์พลังงาน การรีไซเคิล) เป็นเรื่องที่ง่ายดาย (It is easy for me to perform environmentally sustainable activities [e.g., energy conservation, recycling]) *

1 2 3 4 5 6 7

Back | Next

Clear form

สำรวจ / Survey

aj.kevin.fht@gmail.com (not shared) Switch account

* Required

1 = ไม่เห็นด้วยอย่างยิ่ง (Strongly Disagree)
2 = ไม่เห็นด้วย (Disagree)
3 = ค่อนข้างไม่เห็นด้วย (Somewhat Disagree)
4 = เป็นกลาง (Neither Agree or Disagree)
5 = ค่อนข้างเห็นด้วย (Somewhat Agree)
6 = เห็นด้วย (Agree)
7 = เห็นด้วยอย่างยิ่ง (Strongly Agree)

คำถาม 9-14 (Statement 9-14)

9.) ดิฉัน/ผมสามารถควบคุมการกระทำของตนเองเพื่อช่วยเหลือปกป้องสิ่งแวดล้อมได้ (I have control over my actions to support the environment) *

1 2 3 4 5 6 7

10.) มันเป็นการตัดสินใจของดิฉัน/ผมว่าจะปฏิบัติกิจกรรมต่าง ๆ แบบยั่งยืนด้านสิ่งแวดล้อมหรือไม่ (It is my decision whether or not to perform environmentally sustainable activities) *

1 2 3 4 5 6 7

11.) ดิฉัน/ผมมีความสามารถในการดำเนินกิจกรรมต่าง ๆ แบบยั่งยืนด้านสิ่งแวดล้อมได้ (I have the ability to carry out environmentally sustainable activities) *

1 2 3 4 5 6 7

12.) ดิฉัน/ผมสามารถควบคุมตนเองให้ปฏิบัติกิจกรรมต่าง ๆ แบบยั่งยืนด้านสิ่งแวดล้อมได้ (I have control over performing environmentally sustainable activities) *

1 2 3 4 5 6 7

13.) ดิฉัน/ผมวางแผนที่จะเพิ่มกิจกรรมแบบยั่งยืนด้านสิ่งแวดล้อมให้มากขึ้น (เช่น การอนุรักษ์พลังงาน การรีไซเคิล) ในอนาคต (I plan to increase environmentally sustainable activities (e.g., energy conservation, recycling) in the future) *

1 2 3 4 5 6 7

14.) ดิฉัน/ผมตั้งเป้าหมายที่จะค้นหาโอกาสเพื่อการปฏิบัติอย่างแข็งขันทางด้านสิ่งแวดล้อมให้มากขึ้นในอนาคต (I intend to seek out more opportunities to be more environmentally active in the future) *

1 2 3 4 5 6 7

Back | Next

Clear form

สำรวจ / Survey

aj.kevin.fht@gmail.com (not shared) Switch account

* Required

1 = ไม่เห็นด้วยอย่างยิ่ง (Strongly Disagree)
2 = ไม่เห็นด้วย (Disagree)
3 = ค่อนข้างไม่เห็นด้วย (Somewhat Disagree)
4 = เป็นกลาง (Neither Agree or Disagree)
5 = ค่อนข้างเห็นด้วย (Somewhat Agree)
6 = เห็นด้วย (Agree)
7 = เห็นด้วยอย่างยิ่ง (Strongly Agree)

คำถาม 15-20 (Statement 15-20)

15.) ในอนาคต ดิฉัน/ผมวางแผนที่จะค้นหาว่าตนเองจะมีบทบาทในการปกป้องสิ่งแวดล้อมให้มากขึ้นได้อย่างไร (In the future, I plan to look into how I can play a greater role in protecting the environment) *

1 2 3 4 5 6 7

16.) ดิฉัน/ผมไม่ได้คาดหวังที่จะเพิ่มระดับการช่วยเหลือด้านสิ่งแวดล้อมของตนเองให้มากขึ้น (I do not expect to increase my level of support for the environment) *

1 2 3 4 5 6 7

17.) ดิฉัน/ผมพูดถึงความจำเป็นในการอนุรักษ์สิ่งแวดล้อมที่บ้านหรือกับเพื่อน ๆ (I talk about the need to preserve the environment at home or with friends) *

1 2 3 4 5 6 7

18.) ดิฉัน/ผมได้เรียนรู้เกี่ยวกับความยั่งยืนในโรงเรียนมัธยมหรือในมหาวิทยาลัย (I have learned about sustainability in high school or university) *

1 2 3 4 5 6 7

19.) ดิฉัน/ผมได้รับทราบเป็นอย่างดีเกี่ยวกับปัญหาต่าง ๆ ในปัจจุบันที่ส่งผลกระทบต่อสิ่งแวดล้อม (I am well informed about current issues that impact the environment) *

1 2 3 4 5 6 7

20.) ดิฉัน/ผมรู้สึกมั่นใจที่จะพูดคุยเกี่ยวกับประเด็นต่าง ๆ ที่เกี่ยวข้องกับความยั่งยืนด้านสิ่งแวดล้อม I feel confident to talk about issues related to environmental sustainability) *

1 2 3 4 5 6 7

Back | Next

Clear form

สำรวจ / Survey

aj.kevin.fht@gmail.com (not shared) Switch account

นักศึกษาทำแบบสำรวจเกือบเสร็จแล้ว ส่วนนี้เป็นส่วนสุดท้าย (You have answered all the questions of this survey and are almost done)

มีอะไรอื่นอีกหรือไม่ที่นักศึกษาอยากจะแบ่งปัน? (เลือกที่จะตอบหรือไม่ตอบก็ได้) Is there anything else that you would like to share? [optional response]

Your answer

Back | Next

Clear form

สำรวจ / Survey

aj.kevin.fht@gmail.com (not shared) Switch account

ขอบคุณมาก! (Thank you very much!)

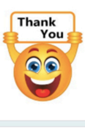

ส่งคำตอบ [Submit Response]

Back | Submit

Clear form
